# P-1406. Evaluating the Diagnostic Significance of Monocyte to Lymphocyte Ratio in diagnosing tuberculosis in HIV infected patients: A Systematic Review

**DOI:** 10.1093/ofid/ofaf695.1593

**Published:** 2026-01-11

**Authors:** Rukesh Yadav

**Affiliations:** Maharajgunj Medical Campus, Institute of Medicine, Tribhuvan University, Kathmandu, Bagmati, Nepal

## Abstract

**Background:**

The blood monocyte-to-lymphocyte ratio (MLR) is associated with active tuberculosis (TB) in adults, but has not been evaluated as a TB diagnostic biomarker in HIV-infected children in whom respiratory sampling is difficult. Clinical paediatric tuberculosis (TB) diagnosis may lead to overdiagnosis particularly among children with human immunodeficiency virus (CHIV). Interferon-gamma release assay and tuberculin skin test use is limited by cost and cross-reactivity with non-tuberculous mycobacteria and Bacille Calmette-Guerin (BCG) vaccination respectively. We assessed the performance of monocyte-lymphocyte ratio (MLR) as a diagnostic biomarker to improve specificity of TB diagnosis in PLHIV with limited access to microbiologic testing.

**Methods:**

We collected data from PubMed, Embase and the Google Scholar. Diagnostic test accuracy studies using MLR to diagnose TB in PLHIV were included. QUADAS tool was used for quality assessment of the included studies.

**Results:**

We screened 4526 publications and included four studies with 3078 participants (adults:2317 and children:761) (age range, children: 0.9 to 6.2 years; mean age range, adult one study mean age 24.5, the other had majority of patients with more than 45 years). All included studies had a high risk of bias. MLR was significantly higher in the HIV patients with TB than those without TB. The cut-off for identification of tuberculosis in PLWHIV among the studies ranged from 0.35 to 0.378. The sensitivity ranged from 12.8 % to 77%, and specificity ranged from 78% to 91.6%. In the study y Naranbhai et al, after adjustment for sex, World Health Organization HIV disease stage, CD4^+^ T-cell counts, and previous history of tuberculosis, hazards of disease were significantly higher for patients with ML ratios of less than the 5th percentile or greater than the 95th percentile (adjusted hazard ratio, 2.47; 95% CI, 1.39–4.40; *P* = .002). After, TB treatment, median MLR declined in children with confirmed TB by Choudhary et al.

**Conclusion:**

The MLR ratio may be a useful, readily available tool to stratify the risk of tuberculosis in PLHIV and further to assess the response to anti-tubercular therapy.

**Disclosures:**

All Authors: No reported disclosures

